# Dual-Polarized Thermal Emission from a Bilayer Twisted-Grating Metasurface

**DOI:** 10.1021/acsphotonics.6c00978

**Published:** 2026-08-02

**Authors:** Xiaohong Zhang, Chiyu Yang, Wenshan Cai, Zhuomin Zhang

**Affiliations:** † School of Electrical and Computer Engineering, 1372 Georgia Institute of Technology, Atlanta, Georgia 30332, United States; ‡ George W. Woodruff School of Mechanical Engineering, 115724 Georgia Institute of Technology, Atlanta, Georgia 30332, United States

**Keywords:** photonic metasurfaces, polarimetry, polarized emitter, thermal radiation, twistoptics

## Abstract

Tailoring thermal radiative properties, including polarization, spectral emissivity, and spatial phase, has attracted an increasing amount of interest in recent years. Polarized thermal emissions can be generated through anisotropic materials, metamaterials, or metasurfaces. Metasurfaces, with the versatile capability to produce differently polarized emissions, have shown significant potential for many advanced photonic applications, such as polarized imaging and chiral molecule sensing. This paper presents a thermal metasurface consisting of two overlapping subwavelength 1D gratings integrated on a single chip, with a relative twist angle that governs the polarization state of the emitted radiation, enabling a transition between dual linear and dual circular polarizations. The spectral emissivity and full set of Stokes parameters of the fabricated metasurface are measured to provide a complete polarimetric characterization. The device exhibits highly polarized, narrowband spectral emission, indicating an enhanced coherence of thermal radiation. The impact of fabrication imperfections on its performance is systematically analyzed. These results highlight the potential of twisted optical metasurfaces for narrowband thermal light sources, infrared imaging, and radiative thermal management applications.

## Graphical abstract

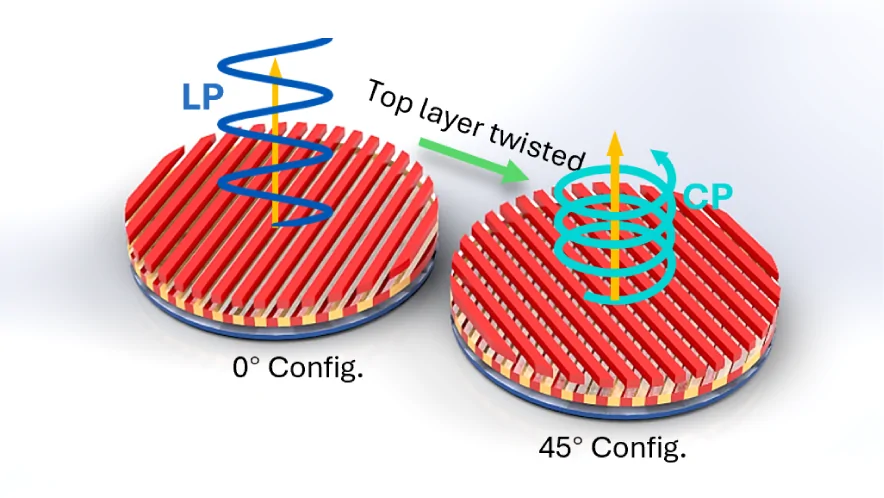

## 1 Introduction

Thermal radiation has been widely utilized in various applications, from everyday appliances to advanced scientific instruments. The origin of thermal radiation can be represented by stochastic current fluctuations within any object above absolute zero, rendering thermal radiation ubiquitous and readily generated.^1,2^ For example, a silicon carbide globar is a simple yet very common choice for a broadband mid-infrared (MIR) light source.^3^ However, because the current fluctuations are highly random and weakly correlated, thermal radiation is traditionally considered incoherent in both space and time, exhibiting a broad emission spectrum, wide angular distribution, and low degree of polarization.^1,4,5^ Nevertheless, a growing number of studies in recent years have shown the feasibility of enhancing the coherence of thermal emission at the structural level, as supported by both theoretical analyses and experiments.^6–11^ Consequently, coherent thermal emitters have emerged as an increasingly attractive research topic.

Highly coherent light sources are essential for emerging MIR technologies, including polarized imaging and remote sensing,^12,13^ polarized/chiral spectroscopy,^14,15^ and narrowband emitters and absorbers.^16–20^ Various approaches have been explored, such as lasers, light-emitting diodes, and metasurface-based thermal emitters. Among them, the quantum cascade laser (QCL) is probably the most prominent MIR source. Despite their excellent coherence, QCLs are limited by high cost, sensitivity to thermal fluctuations, and substantial power consumption.^4^ Meanwhile, thermal emitters inherently exhibit lower coherence; however, partial enhancement in temporal, spatial, or polarization coherence can still meet the requirements of many applications. For example, in application scenarios where phase locking is not required, such as gas sensing, narrow-band thermal emitters provide a practical alternative.^21,22^ Therefore, the development of thermal light sources with enhanced coherence is pivotal from both scientific and engineering perspectives.

Polarization is a fundamental property of light and is closely linked to optical coherence. Highly polarized thermal radiation can be achieved using anisotropic single- or multilayer materials, metamaterials, and metasurfaces.^8,10,23,24^ Thermal metasurfaces, consisting of surface nanostructures on a heated bulk substrate, provide unique radiative properties and emission behaviors that cannot be found in any natural materials. It is worth mentioning that producing polarization-dependent responses using metasurfaces has been widely demonstrated in different wavelength regions.^25–27^ However, most of them are passive and do not actively produce infrared emission. Over the past two decades, the use of active thermal metasurfaces to generate polarized emission in the MIR and far-infrared regions has been advancing rapidly. In 2002, Greffet et al.^9^ experimentally demonstrated linearly polarized (LP) and directional thermal emission for the first time at a wavelength (λ) of ∼11 μm using a silicon carbide subwavelength grating. Yang et al.^28^ later presented a deep aluminum grating capable of producing LP thermal emission at λ ≈ 4 μm. Wang et al.^10^ fabricated an optical antenna metasurface with F-shaped unit cells to produce circularly polarized (CP) thermal emission. Overvig et al.^11^ demonstrated a double-layer thermal metasurface whose polarization can be tuned by modifying the unit-cell geometry. Besides the metasurface approach, polarized thermal emission has also been found in single- or multilayeranisotropic material(s) due to optical birefringence or surface phonon polaritons (SPhPs).^8,23,29–31^ However, the spectral range over which birefringence and SPhPs yield the desired polarization is often fixed by their intrinsic optical properties and is not readily tunable. In comparison, metasurfaces offer greater design flexibility, such as customizable radiation spectra and polarization states, without relying on crystal orientation. Hence, metasurfaces serve as a versatile platform for producing polarized thermal emission in various spectral regions. Exploring thermal metasurfaces with multifunctional and flexible operation is therefore highly promising for advanced photonic applications.

In this work, the design, fabrication, and experimental characterization of a double-layer subwavelength grating metasurface are presented. Two subwavelength gratings with distinct functionalities are designed to be overlapped and rotated against each other in-plane to form a “twisted-grating” structure; such a geometry effectively modifies the lattice without altering the structure of individual unit cells. The polarization state is determined by the designed relative twist angle between the two grating layers. For a proof-of-concept demonstration, two configurations with fixed twist angles, 0° and 45°, were fabricated to produce emission with linear polarization (LP) and circular polarization (CP), respectively. Because this approach does not require changes to the geometric dimensions within each unit cell except for the twist angle, it offers a potential route toward reconfigurable thermal emitters if combined with mechanical in-plane rotation through microelectro-mechanical systems (MEMS) actuation. To fully understand and characterize the twisted gratings, the complete set of Stokes parameters was measured and analyzed using a polarized infrared emissometer. In the experiment, strong LP emission was observed for the 0° configuration, whereas relatively weak CP emission was observed for the 45° configuration, presumably due to fabrication imperfections in both grating layers. The impact of fabrication errors on device performance is numerically analyzed to explain the measurement results and identify ways for future improvement. Overall, this work demonstrates a simple and effective approach to generating polarized thermal emission and advances thermal metasurfaces toward multifunctional operation.

## 2 Design and Working Principles

The proposed twisted-grating metasurface consists of two layers of amorphous silicon (a-Si) gratings and an aluminum back reflector integrated on a single chip, as shown in Figure 1. The top a-Si grating and the SiO_2_ spacer are designed to serve as an effective quarter-wave plate. The bottom a-Si grating is filled with copper to form a diluted metal layer and provides polarized emission through structural anisotropy. Copper is selected for its relatively high surface mobility during electron-beam evaporation and low cost. An SiO_2_ layer is placed between the grating layers to eliminate interlayer coupling and control phase retardation, and another SiO_2_ layer is deposited between the bottom grating and the aluminum reflector to enhance emissivity via cavity resonance. The device supports two polarization states depending on the relative twist angle of the top grating. When the grooves of the top and bottom gratings are aligned (Figure 1a), the emission is linearly polarized. When the two gratings form a relative angle of 45° (Figure 1b), the emission becomes highly circularly polarized. These two states are denoted as the “0° configuration” and the “45° configuration”. The geometric parameters were optimized using a particle swarm optimization algorithm as implemented in the Scikit-Opt Python package^32^ then manually verified. Details about the optimization process and the figure-of-merit are included in the Supporting Information. The final geometric dimensions are shown in Table 1.

**Figure 1:**
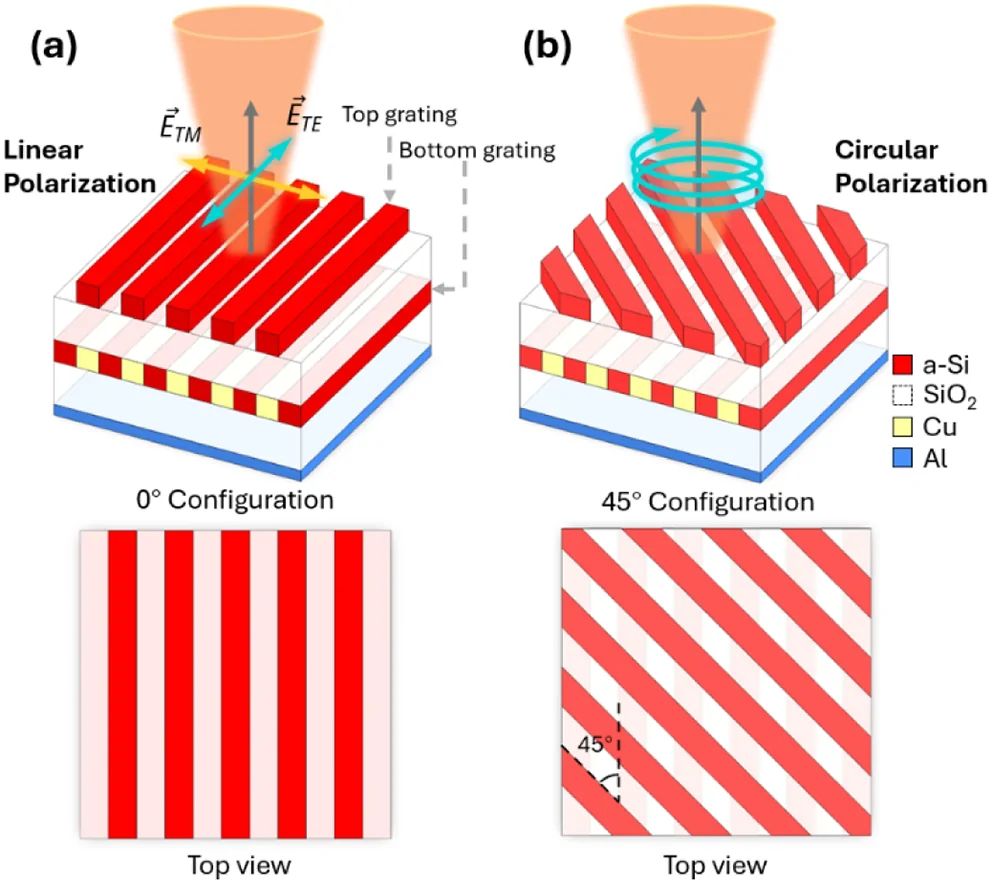
Overview of the twisted-grating metasurface. Representative unit cells spanning multiple grating periods are shown, with each material indicated by the colormap on the right. (a) 0° configuration, in which the top and bottom gratings are aligned, producing linearly polarized emission upon heating. The transverse electric (TE) and transverse magnetic (TM) directions are indicated by the orange and cyan arrows, respectively. (b) 45° configuration, in which the top grating is rotated by 45° relative to the bottom grating, designed to generate left- and right-handed circularly polarized emission (LCP and RCP).

**Table 1:** Geometric Dimension of the Twisted Grating Metasurface as Named in Figure 1 ^a^

Structure	Thickness (μm)	Period (μm)	Fill Factor
Top grating	0.9	2.0	0.6
Bottom grating	0.87	2.0	0.5
Top oxide	1.95	N/A	N/A
Bottom oxide	2	N/A	N/A

a Note that the 0° and the 45° configurations share the same geometry, except for the angle of the top grating .

The directional-hemispherical reflectivity for different polarizations at normal incidence is calculated using finite-difference time-domain (FDTD) method. The spectral polarized emissivity can then be obtained using Kirchhoff’s law:^1^

1 $$\epsilon_{i,\lambda }=\alpha_{i,\lambda }=1-\rho_{i,\lambda }$$

where *i* implies the polarization states, defined in either the TE/TM basis or the LCP/RCP basis. Here, *ϵ*_*i*,λ_, *α*_*i*,λ_, and *ρ*_*i*_,_λ_ represent the emissivity, absorptivity, and reflectivity at wavelength λ in the normal direction, respectively. The transmission term vanishes due to the aluminum back reflector, which is thick enough to eliminate transmission. The polarized emissivity spectra of the two device configurations were obtained using FDTD simulation and are shown in Figure 2. For the 0° configuration, a TM and a TE resonance occur at 5.13 and 5.4 μm, respectively, while for the 45° configuration, LCP and RCP resonance peaks occur at nearly the same wavelengths.

**Figure 2:**
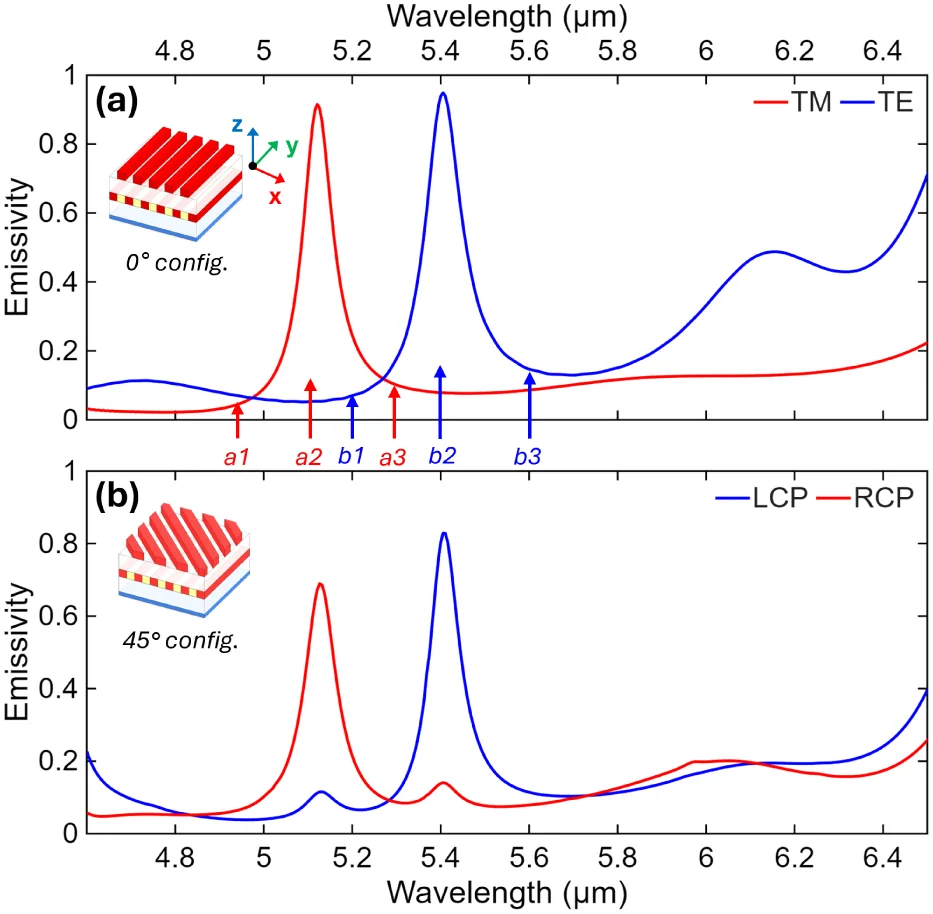
FDTD simulation results of polarized emissivities for different rotation angles of the top grating. (a) The LP spectral emissivity of the 0° configuration. The wavelengths are marked by a1 = 4.9 μm, a2 = 5.1 μm, a3 = 5.3 μm, b1 = 5.2 μm, b2 = 5.4 μm, and b3 = 5.6 μm. (b) The CP spectral emissivity of the 45° configuration, showing a high degree of circular polarization at the same wavelengths, a_2_ and b_2_.

To further elucidate the function of each layer, electric field distributions at the wavelengths marked in Figure 2a are shown in Figure 3 for both linear polarizations of the 0° configuration. Following the definition of TE (*E*_y_) and TM (*H*_y_) modes in Figure 2, the dominant field components of each polarization (i.e., *E*_y_ for TE and *E*_x_ for TM) are considered and shown in Figure 3. Other field components are relatively small compared to the main components and therefore are not shown. The electric field distributions indicate that a Fabry-Pérot cavity coupled with a grating structure gives rise to the emissivity peaks. The cavity consists of the bottom SiO_2_ layer between a top leaky metallic grating and a back aluminum layer. At the resonant wavelengths (a_2_ and b_2_), strong and spatially uniform electric field confinement is observed within the oxide layer, whereas the field intensity is significantly reduced off resonance. The subwavelength grating structure on top of the bottom oxide can be treated as a diluted metal with strong anisotropy, which can be described by effective medium theory.^33,34^ It is worth noting that the polarization effect in such a cavity is due to the different polarization selectivity of the grating atop, and thus leads to distinct resonances for different polarizations. In contrast, the top grating in the 0° configuration has a negligible effect on polarization. As can be seen in Figure 3, there is no significant electric field or resonance of any kind induced inside the top grating region throughout the entire wavelength range of interest.

**Figure 3:**
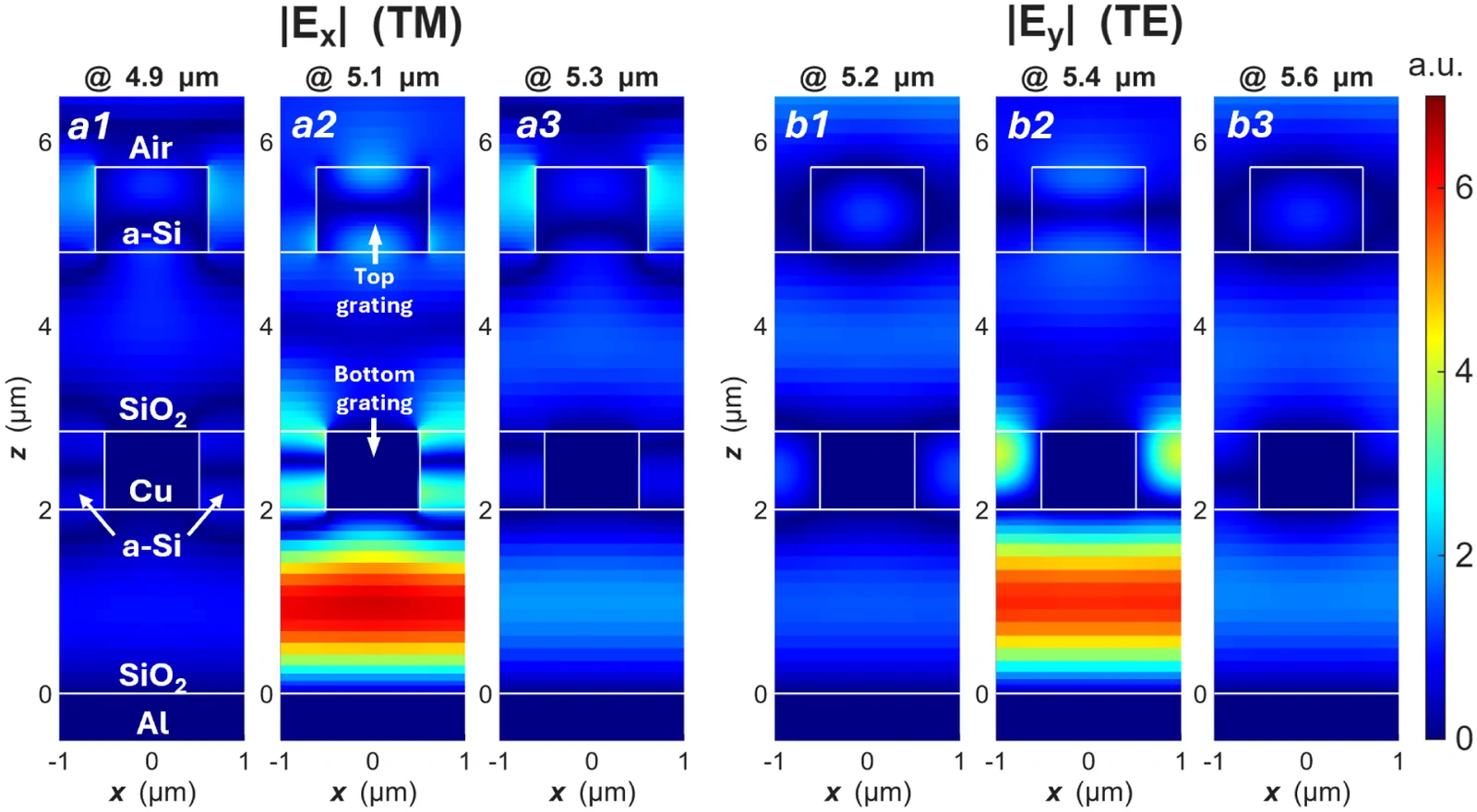
Electric field amplitude distributions, in arbitrary units (a.u.), of |*E*_x_| for the TM mode and |*E*_y_| for the TE mode at the wavelengths marked by a1, a2, etc. in Figure 2. Note that a2 and b2 corresponds to the resonances, and a1, a3, b1, and b3 are off-resonance. The plot shows the cross-sectional profile of the *xz*-plane. The white contours marks the boundaries of each material.

Additional RCWA simulations were performed to further elaborate on the function of each individual grating layer and to investigate the device’s emissivity, *ϵ*, at oblique angles, as shown in Figure 4. Two cases were considered: (1) a structure with only the bottom grating and (2) the entire structure with both (aligned) grating layers. Different contour lines for oblique angles are determined by varying the amplitude of the transverse wavevector ***k***_x_. The light line represents the dispersion of light in the vacuum, *ω* = *ck*_0_, which also corresponds to the 90° limiting case, *k*_x_ = *k*_0_. For the structure containing only the bottom grating, as shown in Figure 4a,b, strong linearly polarized emissivity peaks are observed at the target wavelengths, confirming that the bottom grating and everything beneath it are the source of linear polarization. The peaks near *λ =* 5.4 μm for TE and *λ =* 5.1 μm for TM remain nearly unchanged up to an emission angle of about 20°. The subsequent shift of the resonant wavelength with respect to the increasing emission angle is a common phenomenon in grating structures due to changes in the phase-matching condition. In practice, the spatial components containing undesired wavelengths can be filtered out by using an iris to restrict the emission angle. For the complete structure with two grating layers, as shown in Figure 4c,d, the emission peaks are attenuated by approximately 10–20% across the spectrum due to additional losses and parasitic modes introduced by the top grating. The positions of the previous emissivity peaks in the wavelength spectrum, however, are nearly unchanged in the 0° configuration, as mentioned previously. When the top grating is rotated by 45°, the emission transitions from linear to circular polarization, as discussed previously. These results indicate that the top grating functions as an integrated waveplate with its slow axis aligned along the grooves.

**Figure 4:**
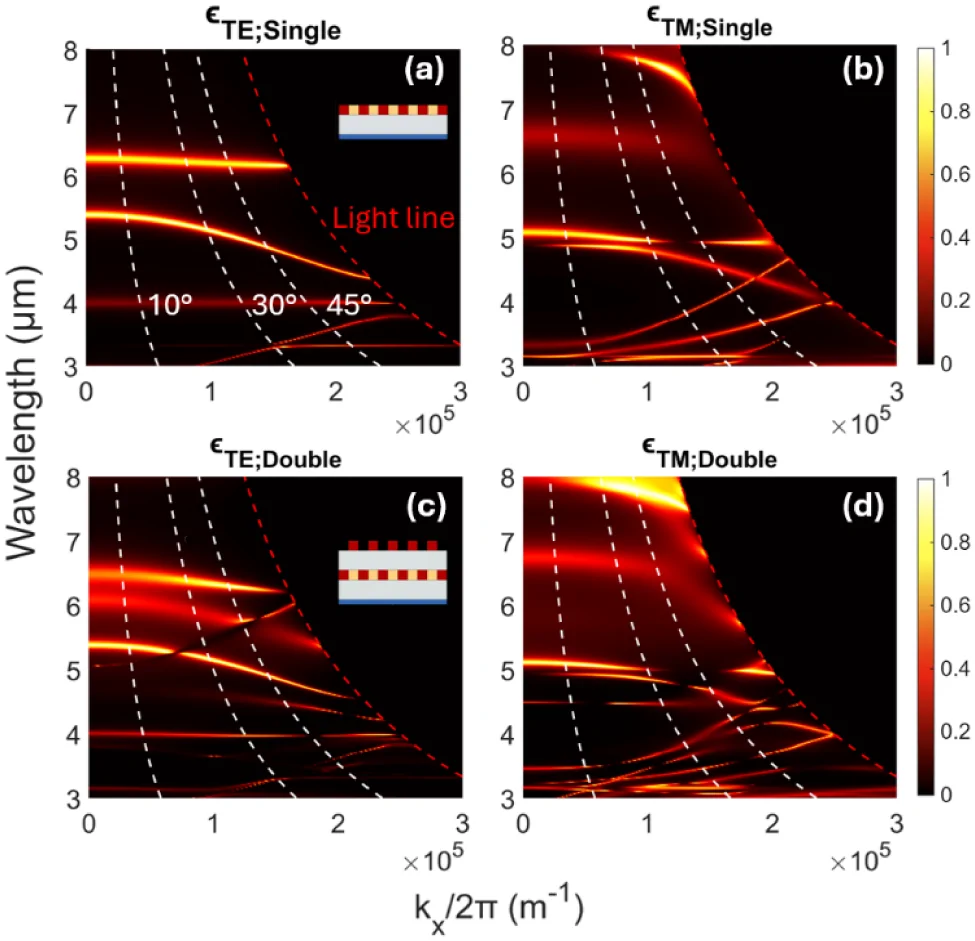
RCWA-simulated emissivity dispersion of the bottom grating (*ϵ*_Single_; a and b) and the complete bilayer structure (*ϵ*_Double_; c and d). Contours corresponding to different oblique emission angles are shown in white, and the light line is indicated in red. The bottom grating exhibits strong linearly polarized resonances in the 5–7 μm range, confirming its role as an effective linear emitter.

The emissivity of the 45° configuration under oblique incidence was also obtained using FDTD by simulating the reflectance and absorptance and using eq 1, as shown in Figure 5. Using the coordinate convention in Figure 2a, the angle of incidence is defined as the elevation angle in the *xz*-plane with respect to the *z*-axis and rotates toward positive *x*. For LCP, the peak emissivity moves from 5.4 to 5.37 μm at 10° and 5.16 μm at 30°. For RCP, the peak emissivity moves from 5.13 to 5.09 μm at 10° and 4.87 μm at 30°. The drift of the resonance wavelength is 4.4% for LCP and 5% for RCP.

**Figure 5:**
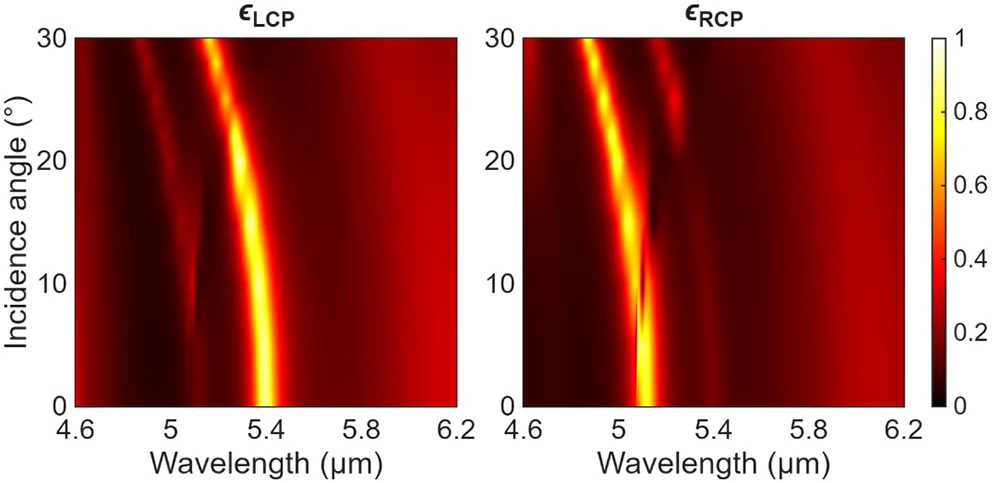
Response of the 45° configuration under oblique incidence for (a) LCP and (b) RCP, based on FDTD simulations. The angle of incidence is defined as the elevation angle in the *xz*-plane with respect to the *z*-axis, following the coordinate system shown in Figure 2a.

## 3 Device Fabrication

The fabrication process for the twisted gratings is outlined in Figure 6. The aluminum back reflector is first deposited on a Si substrate by electron-beam evaporation, followed by SiO_2_ and a-Si layers deposited by plasma-enhanced chemical vapor deposition (PECVD). The a-Si film contains hydrogen bonds (a-Si:H), and its refractive index is characterized by Fourier transform infrared spectroscopy (FTIR) (see the Supporting Information). Grating patterns are defined using photolithography to enable higher throughput compared with electron-beam lithography, which is commonly employed in photonic device fabrication.^35^ After that, the a-Si layer is etched by inductively coupled plasma (ICP) etching to form the desired trench structures. To realize the interleaving pattern of Cu and a-Si, the trenches were filled with copper deposited by electron-beam evaporation. The deposition time is precisely controlled to achieve planar filling up to the top surface of the a-Si layer, after which excess metal is released and removed along with the photoresist. The subsequent fabrication steps repeat the previous steps, including a very similar thin-film deposition, photolithographic patterning, and plasma etching. Two configurations of the device were made separately. After fabrication, scanning electron microscopy (SEM) images of the device are taken, as shown in Figure 7. Most geometric features are well presented in the fabricated device, except for the Cu-a-Si interleaving pattern, which exhibits slightly imperfect filling due to the limitations of electron-beam evaporation. Such a gap defect has a pronounced influence on the performance of the device and is shown in the next section.

**Figure 6:**
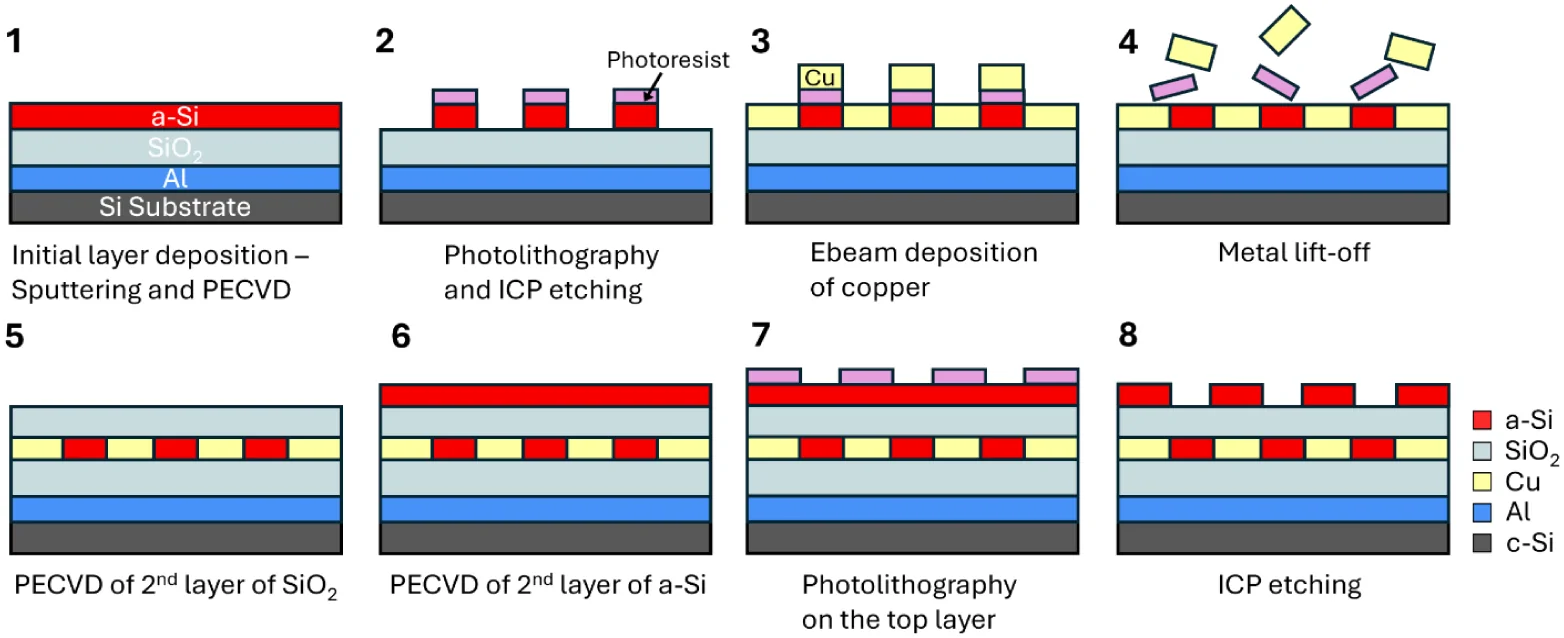
Fabrication process of the twisted-grating metasurface. A photolithography scanner with the highest resolution of 600 nm was used. A combination of electron-beam deposition and liftoff was used for metal trench filling in the first grating layer.

**Figure 7:**
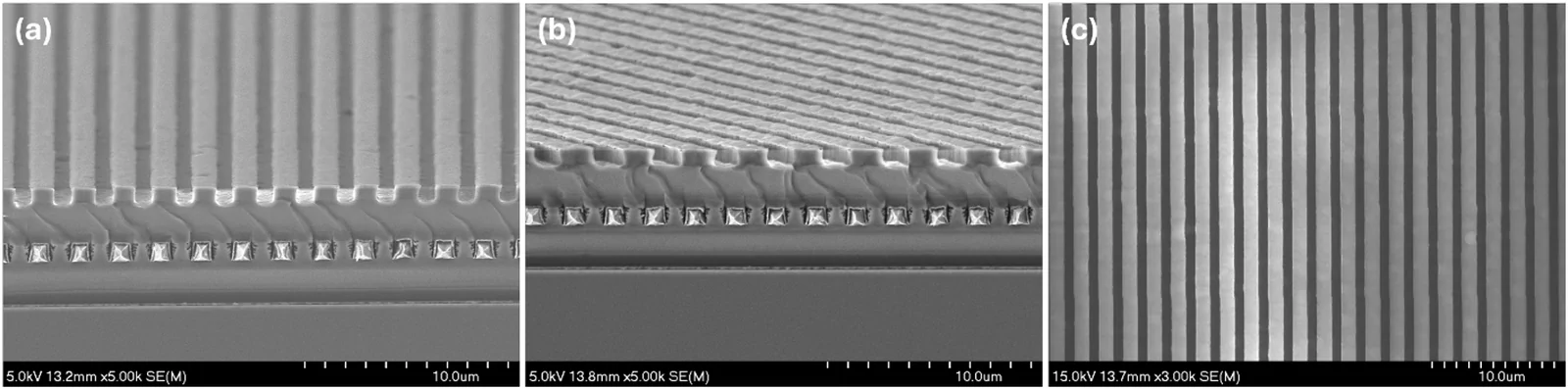
SEM photos of the devices. (a) Cross-sectional profile of the metasurface with 0° configuration. (b) Cross-sectional profile of the metasurface with 45° configuration. (c) Top view of the top grating.

## 4 Measurement Results

To measure the emissivity of the metasurface, a polarized emissometer was developed based on previous works.^36–38^ The overview of the setup is shown in Figure 8, with additional details provided in the Supporting Information. The experimental setup contains three main parts: (1) the sample is placed on a resistive heater inside a vacuum chamber, which has a zinc selenide (ZnSe) optical window on its sidewall. The chamber creates a low-pressure environment that minimizes temperature fluctuations from air convection and protects the device. (2) The polarization analyzer consists of a ZnSe Fresnel rhomb (which functions as an achromatic waveplate) and a linear polarizer. (3) A pair of mirrors, consisting of an ellipsoidal mirror and a parabolic mirror, collects and collimates the beam and directs it into the FTIR.. For circular emissivity measurements, the emitted light first passes through the Fresnel rhomb, which converts the circularly polarized components into 45° and 135° linearly polarized components. These components are then selected by setting the linear polarizer to 45° or 135°. For linear polarization measurements, only the linear polarizer is used, and the Fresnel rhomb is removed. The entire optical setup is realigned afterward.

**Figure 8:**
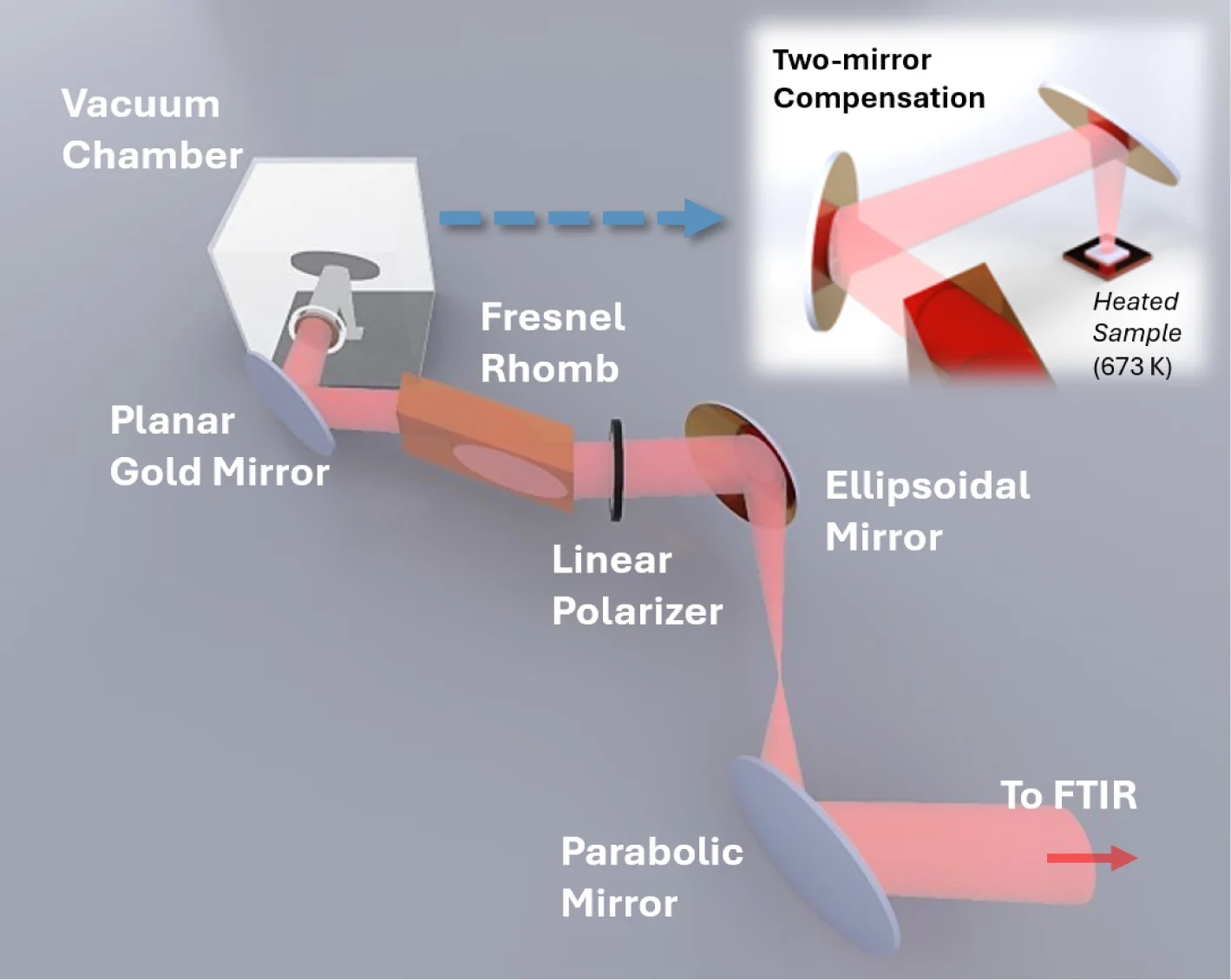
Schematic of the measurement setup. A two-mirror method is employed to eliminate the circular dichroism induced on each mirror. The Fresnel rhomb functions as an achromatic waveplate and, together with a linear wire-grid polarizer, serves as a CP analyzer. Nitrogen purging is applied to the entire setup (not shown).

The temperature of the sample is set to 673 K (400 °C), with temperature variations maintained below 0.5 K. The heater is made of high-purity copper to improve temperature uniformity and minimize its own emission. The whole optical setup is enclosed in a nitrogen-purged environment to minimize atmospheric absorption in the MIR range.

The measurement results of polarized emissivity are shown in Figure 9a,b,d,e. The Stokes parameters are shown in Figure 9c,f. Polarized emissivity, *ϵ*_λ,_ *_i_*, is defined as the ratio of the emitted power with respect to a blackbody reference:

**Figure 9:**
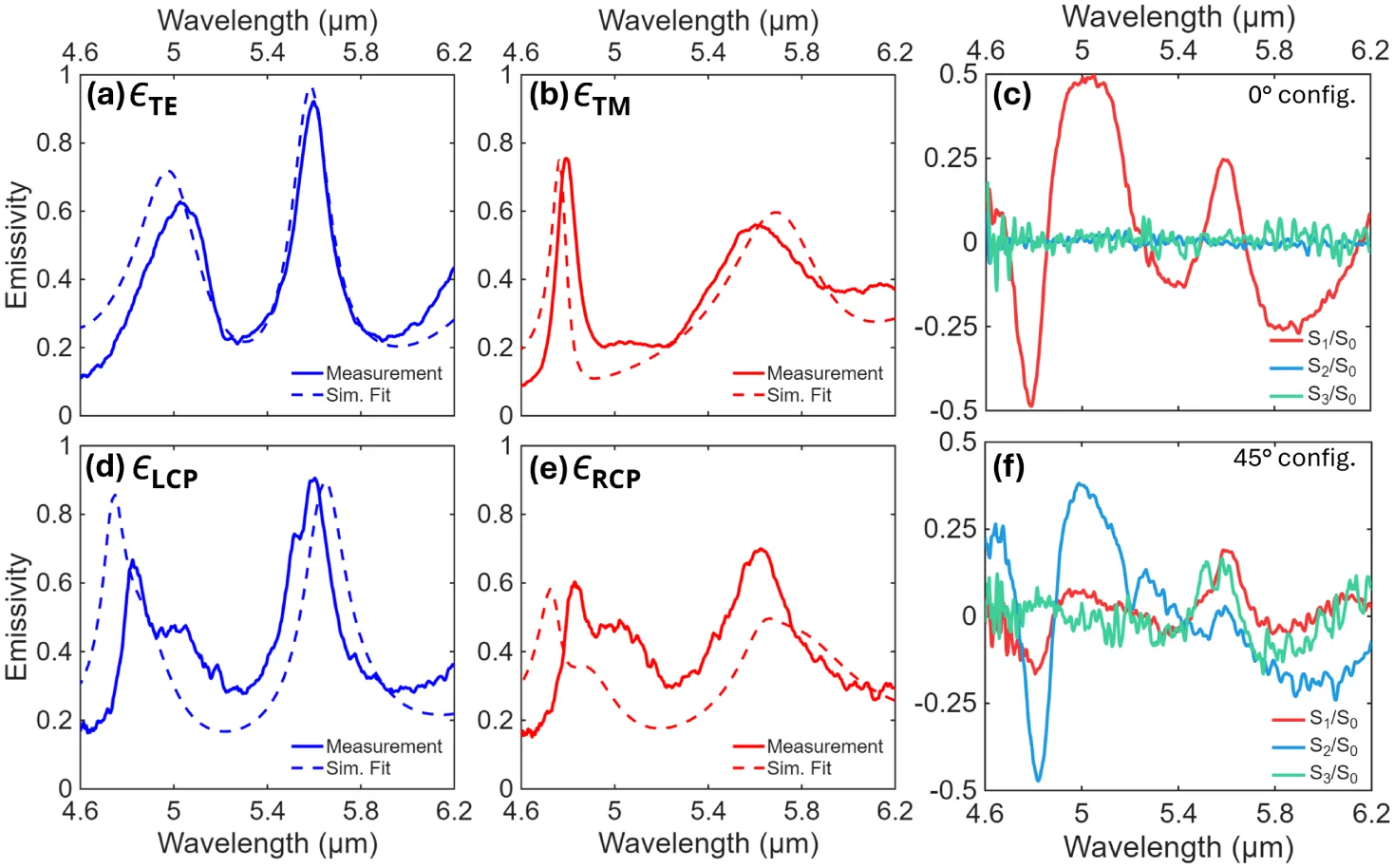
(a) and (b) Measured and simulation-fitted linearly polarized emissivities and (c) normalized Stokes parameters for the 0° configuration. (d) and (e) Measured and simulation-fitted circularly polarized emissivities and (f) normalized Stokes parameters for the 45° configuration. A four-point moving averaging was applied to reduce noise in the data.


2 $$\epsilon_{\lambda ,i}=\frac{I_{\lambda ,i}^{\mathrm{DUT}}}{I_{\lambda ,i}^{\mathrm{REF}}}$$

where $I_{\lambda ,i}^{\mathrm{DUT}}$ is the spectral intensity of the received signal from the device under test (DUT), and $I_{\lambda ,i}^{\mathrm{REF}}$ is the reference blackbody. In the measurement, a specimen of vertically aligned carbon nanotubes (CNTs) is used as a reference, whose emissivity has been verified to be greater than 0.995 in the MIR according to a previous publication.^39^ From Figure 9a,b, for the 0° configuration, a TE emissivity of 92% and a TM emissivity of 75% are observed at 5.6 and 4.8 μm, respectively, with a slight shift from the designed wavelengths due to fabrication uncertainties. For the 45° configuration, the emissivity of the device is 90% and 69% for LCP and RCP at 5.6 μm, as shown in Figure 9d,e. The circular dichroism (*ϵ*_λ,LCP_ – *ϵ*_λ,RCP_) is approximately 21%. The degradation of the degree of circular polarization is due to the fabrication imperfections, especially the imperfect filling of the trenches in the bottom grating that creates air gaps between the a-Si and Cu in the bottom grating. From the SEM images (Figure 7a,b), the air gap between a-Si and copper is estimated to be around 180 nm and partially filled with SiO_2_ from the layer above. It should be emphasized that the electromagnetic boundary conditions, especially for TM mode, of perfectly filled trenches in the bottom grating differ significantly from those of partially filled structures, particularly when the residual gaps are not negligibly small relative to the feature size. In the ideal case, the a-Si and copper contact seamlessly, whereas in the fabricated sample, an air gap separates the two materials. This discontinuity alters the boundary conditions and consequently modifies the modal profile, causing the performance to degrade. Future improvements, such as incorporating spin-on glass for grating formation, may mitigate these issues and enable more accurate fabrication with improved polarization performance. Additional simulations were therefore performed to investigate how layer-thickness variations, air-gap width, and variations in the refractive index of a-Si affect the device performance. Compared with the other factors, whose effects are shown in the Supporting Information, the air gap has a relatively larger impact on the device performance, particularly for circular polarization, and thus is explicitly shown in Figure 10. As the air-gap width increases, the LCP peak shifts toward longer wavelengths, whereas the RCP peak shifts toward shorter wavelengths. In addition, the RCP peak amplitude decreases significantly with increasing air-gap width, which partially explains the diminished RCP emissivity observed in the measured results. However, it should be noted that the measured spectra contain additional unexpected features that cannot be fully explained by the air-gap defect alone. These features can be more consistently accounted for only by considering other factors together, although the air gap remains the dominant factor affecting the overall device performance.

**Figure 10:**
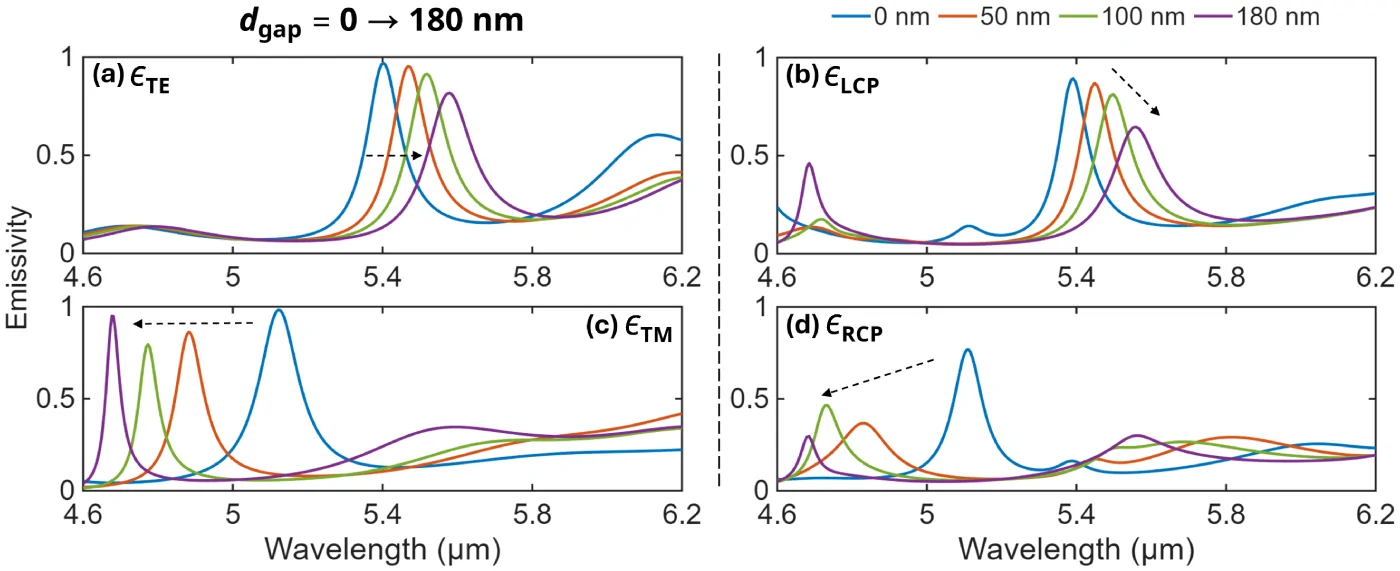
Impact of air gap defects on the polarized emissivities of the device for (a) TE, (b) LCP, (c) TM, and (d) RCP, obtained from FDTD simulations. The width of the gap ranges from 0 to 180 nm. The air gap is set to occupy the space of Cu. All other parameters remain at their original values.

The fitted simulation results are shown in Figure 9a,b,d,e as dotted curves. The parameters used in the simulation are adjusted to fit the experiments, namely, the top grating thickness is 1070 nm, the bottom grating thickness is 980 nm, the air gap width is 180 nm in the bottom grating, which reduces the width of Cu ridges, and the modified a-Si optical constants are *n* = 3.28 and *κ*=0.03 at 5 μm. The air gaps occupy the space of Cu because they are caused by imperfect trench filling during electron-beam deposition.

To further characterize the polarization state of the metasurface, the Stokes parameters, as defined in terms of polarized emissivities, were measured.^34,36^

3 $$\{\begin{array}{l} S_{0,\lambda }=\epsilon_{\lambda ,0^{{}^{\circ}}}+\epsilon_{\lambda ,90^{{}^{\circ}}}=\epsilon_{\lambda ,\mathrm{LCP}}+\epsilon_{\lambda ,\mathrm{RCP}}\\ S_{1,\lambda }=\epsilon_{\lambda ,0^{{}^{\circ}}}-\epsilon_{\lambda ,90^{{}^{\circ}}}\\ S_{2,\lambda }=\epsilon_{\lambda ,45^{{}^{\circ}}}-\epsilon_{\lambda ,135^{{}^{\circ}}}\\ S_{3,\lambda }=\epsilon_{\lambda ,\mathrm{LCP}}-\epsilon_{\lambda ,\mathrm{RCP}} \end{array}$$

In eq 3, *ϵ_λ_*,_*θ*_ denotes the emissivity for the polarization angle of *θ* measured with respect to the horizontal *x*-axis. Consistent with the notation used in the previous context, the TE wave *ϵλ,*TE = *ϵλ,*_0°_ and the TM wave *ϵ_λ_*,_TM_ = *ϵ_λ_*,_90°_. It should be noted that the Stokes parameters are defined differently here using polarized emissivities rather than absolute intensities. Therefore, parameter *S*_0_ is related to, but does not directly represent, the total intensity of the emission. The remaining Stokes parameters represent the polarization of the emissivity for each orthogonal polarization pair. It is worth noting that the measurement of *S*_1_ and *S*_3_ requires temporarily removing and reintroducing the Fresnel rhomb, which translates the light beam, from the optical path. In addition, the FTIR has a polarization-dependent response, which must be considered in the postprocessing of the measurement data.^36^ Therefore, to obtain the correct degree of polarization, the measured light intensity needs to be normalized to the corresponding intensity of the reference sample for each polarization, respectively. This normalization preserves the relative values of the Stokes parameters by accounting for the polarization and spectral response of the setup. It should also be pointed out that, although the linear and circular measurement setups have different losses and therefore different measured light intensities, the *S*_0_ stays the same for both, i.e., *ϵ_λ_*,_0°_ + *ϵ_λ_*,_90°_ = *ϵ_λ_*,_LCP_ + *ϵ_λ_*,_RCP_, because the setup-dependent loss is largely canceled after taking the intensity ratio for the emissivity. Thus, the summation should be the same when switching the bases between LP and CP. Further details about the measurement are included in Supporting Information.

The measured normalized Stokes parameters shown in Figure 9c suggest that, for the 0° configuration, *S*_1_/*S*_0_ is −0.48 at 4.8 μm and 0.24 at 5.6 μm, while *S*_1_/*S*_0_ and *S*_3_/*S*_0_ are close to zero at both wavelengths. This result suggests predominantly linear polarization, as the *S*_1_/*S*_0_ is much larger than *S*_2_/*S*_0_ and *S*_3_/*S*_0_, although a substantial unpolarized component is also present. For the 45° configuration, whose measured Stokes parameters are shown in Figure 9f, *S*_3_/*S*_0_ remains small across most of the spectrum but reaches 0.17 at 5.6 μm, indicating partial LCP. At 4.8 μm, however, the *S*_3_/*S*_0_ is nearly zero, while strong *S*_2_/*S*_0_ can be observed, indicating that the polarization state of this device is close to 135° linear polarization. This deviation from the intended circular polarization in the simulation is primarily attributed to fabrication imperfections, particularly incomplete trench filling in the bottom grating, as mentioned before.

## 5 Conclusion

In this work, a twisted-grating thermal metasurface operating in the MIR is presented. The design employs two configurations with different top-grating twist angles to select either linear or circular emission states. The two grating layers serve different functions, with the bottom grating acting as part of the linear emitter and the top one functioning as an effective waveplate. A polarized infrared emissometer with sample temperature and pressure control is developed to measure the device’s polarized emissivity and Stokes parameters at 673 K.

The measured emissivities of the 0° configuration device are 75% for TM polarization at 4.8 μm and 92% for TE polarization at 5.6 μm. The absolute emissivity differences between the two polarizations at these wavelengths are 50% and 38%, respectively, indicating the presence of unpolarized emission components. The corresponding Stokes parameters further confirm that the polarized component is predominantly linearly polarized, with negligible circular or diagonal linear components. For the 45° configuration device, the emissivity reaches 69% for RCP and 90% for LCP at 5.6 μm. However, the circular dichroism is limited to approximately 21% due to fabrication imperfections, primarily incomplete trench filling. The Stokes parameters indicate that the emission is nearly linearly polarized at 4.8 μm and elliptical at 5.6 μm. Potential improvements to the fabrication process include refining trench filling and planarization, such as using chemical mechanical polishing or further simplifying the structure and using spin-on glass for the double-layer grating. This work provides evidence for the feasibility of using a twisted bilayer subwavelength grating to create potentially reconfigurable thermal metasurfaces if combined with MEMS or other mechanical actuation mechanisms. This platform may have potential applications in polarized thermal imaging, chemical sensing and spectroscopy, and radiative heat transfer.

## Supplementary Material



## Notes

Additional experimental details and discussions on the effect of fabrication imperfections and the optimization process (PDF)
