# Supplementary material for: Dual-Polarized Thermal Emission from a Bilayer Twisted-Grating Metasurface
Source: ACS Photonics. 2026 Aug 2;13(16):4726–34. doi: 10.1021/acsphotonics.6c00978 (PMC13495958; doi:10.1021/acsphotonics.6c00978)
Supplement: Supplementary file 1 [file ph6c00978_si_001.pdf]

Supporting Information for

Dual-Polarized Thermal Emission from a Bilayer Twisted-Grating Metasurface

Xiaohong Zhang,[1] *Chiyu Yang,*[2] *Wenshan Cai,*[1] *and Zhuomin M. Zhang**[2]

[1]School of Electrical and Computer Engineering, Georgia Institute of Technology, Atlanta, Georgia 30332, USA

[2]George W. Woodruff School of Mechanical Engineering, Georgia Institute of Technology, Atlanta, Georgia 30332, USA

*E-mail: zhuomin.zhang@me.gatech.edu

Experimental setup; Refractive index measurement of a-Si; Effect of the fabrication imperfections; Optimization Process

Pages S1-S12; Figures S1-S5

Experimental setup

The optical path is shown in Figure S1a, and the components inside the vacuum chamber are covered in Figure 7a in the main text. The entire setup contains three main parts. First, the diced sample, with an active device area of 1 cm × 1 cm, is placed on a customized sample holder with a pre-cut slot (Fig. S1b). Copper is selected for its great thermal conductivity and low emissivity. Non-bonding boron nitride thermal paste featuring a thermal conductivity of 31.4 $W \cdot m^{-1} \cdot K^{-1}$ is used to ensure effective conduction between the heated sample holder and the sample. A thin copper disk with a square aperture is placed and secured atop the sample to limit the radiating area. Then, the copper sample holder is placed on a resistive heater (MHI Inc.)[1]. A thermocouple for monitoring the temperature is inserted into the designed slot on the sample holder, and the tip of the thermocouple is right beneath the position of the sample. Thermal paste is also applied between the sample holder and the heater, and between the thermocouple and the sample holder. The whole

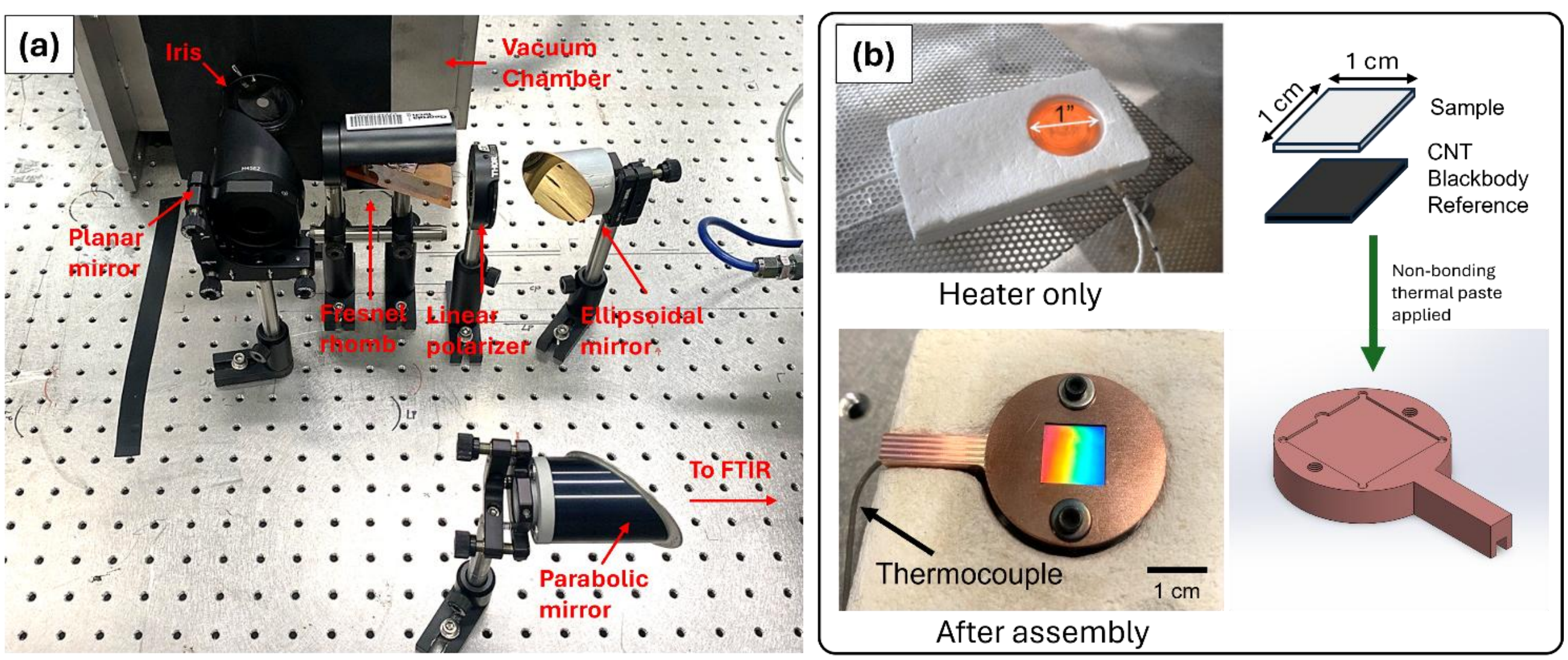

Figure S1. Measurement setup for circular polarized emissivity. (a) The optical setup. For the complete setup including the components inside the chamber, please refer to Figure 7a in the main text. (b) Sampler heater and its parts. The upper left picture is taken from the website of MHI Inc.[1]

assembly is placed inside a stainless-steel vacuum chamber. Temperature control is via a PID control algorithm which reads the temperature from the thermocouple and controls the output of a DC power supply. In the experiment, the sample is heated up first to 473 K then 673 K to avoid thermal shock and drastic change of the chamber pressure. The measurements are performed after the chamber pressure reaches below 0.01 Pa, where the atmospheric absorption becomes negligibly small.

Because the heater is placed horizontally with the sample facing up, a gold planar mirror without any protective coating (customized from Thorlabs, Inc.) is used to reflect the beam. In practice, the gold mirror still has moderate circular dichroism and needs to be taken into consideration. Therefore, a second identical same mirror, rotated by 90° to swap the TE and TM components, is placed outside the chamber to eliminate the circular dichroism introduced by a single mirror. The light then passes through a horizontally slanted Fresnel rhomb and a linear polarizer at 45°/135°. This combination distinguishes the light intensity of the two circular polarization states, and S_0 and S_3 can be measured by using the classical Stokes parameters measurement approach. Note that when measuring the linear polarizations (S_1 and S_2), the Fresnel rhomb is removed from the optical path, then the entire setup is re-aligned. The measured light intensity is normalized to the intensity of the reference sample for the corresponding polarization and setup, which is denoted as $I_{\lambda,\mathrm{TE/TM}}^{\mathrm{REF}}$ and $I_{\lambda,\mathrm{LCP/RCP}}^{\mathrm{REF}}$. Therefore, all polarization and spectral response of the measurement system are cancelled when calculating the emissivity, as follows:

$$\epsilon_{\lambda,i} = \frac{I_{\lambda,i}^{\mathrm{DUT}}}{I_{\lambda,i}^{\mathrm{REF}}} = \frac{\mathcal{R}_{\lambda,i}\epsilon_{\lambda,i}^{\mathrm{DUT}} I_{\lambda,i}^{\mathrm{bb}}}{\mathcal{R}_{\lambda,i} I_{\lambda,i}^{\mathrm{bb}}} = \epsilon_{\lambda,i}^{\mathrm{DUT}}, \tag{S1}$$

where $\mathcal{R}_{\lambda,i}$ represents the spectral polarized response of the measurement setup for the corresponding polarization state *i*, including the different loss for linear and circular measurement, polarized response of the FTIR, and detectors. $I_{\lambda,i}^{\mathrm{bb}}$ is the intensity of an ideal blackbody, and the reference sample is assumed to have a unit emissivity. By using the emissivity instead of the absolute intensity, the systematic bias of the measurement system is largely cancelled.

The corresponding degree of polarization values are obtained through the following:

$$\begin{cases} S_{0,\lambda} = \epsilon_{\lambda,0^\circ} + \epsilon_{\lambda,90^\circ} = \epsilon_{\lambda,LCP} + \epsilon_{\lambda,RCP} \\ S_{1,\lambda}/S_{0,\lambda} = \frac{\epsilon_{\lambda,0^\circ} - \epsilon_{\lambda,90^\circ}}{\epsilon_{\lambda,0^\circ} + \epsilon_{\lambda,90^\circ}} \\ S_{2,\lambda}/S_{0,\lambda} = \frac{\epsilon_{\lambda,45^\circ} - \epsilon_{\lambda,135^\circ}}{\epsilon_{\lambda,0^\circ} + \epsilon_{\lambda,90^\circ}} \\ S_{3,\lambda}/S_{0,\lambda} = \frac{\epsilon_{\lambda,\mathrm{LCP}} - \epsilon_{\lambda,\mathrm{RCP}}}{\epsilon_{\lambda,\mathrm{LCP}} + \epsilon_{\lambda,\mathrm{RCP}}} \end{cases} . \qquad (S2)$$

The whole optical setup is placed in a nitrogen gas purge box to minimize the atmospheric absorption in the mid-IR range. An FTIR (ABB FTLA-2000) is used for scanning the emission over the spectrum at a resolution of 2 cm^{-1}. To reduce the noise, 256 scans are taken for each measurement.

<u>Refractive index measurement of a-Si</u>

The PECVD deposition of a-Si includes SiH_4 and H_2 gases, and hydrogen bonds can form within the deposited layer, i.e., hydrogenated a-Si (a-Si:H). The hydrogen bonds change the optical properties of a-Si depending on the H_2 flow rate in the process. Therefore, it is necessary to measure the refractive index of the a-Si and tune the design accordingly. Two a-Si samples (0.5 μm and 1 μm thick) deposited on a 1-mm thick SiO_2 substrate are used for the measurement. The

same FTIR is used for measuring the reflectance of the sample at 10° incident angle to minimize the polarization dependency, while a gold reference is used to provide a reflectance reference. Considering the strong absorption of SiO_2 beyond 5 μm, the theoretical reflectance is modeled by a thin-thick layer model when the wavelength $\lambda < 5$ μm and a simple multiple-reflection model when $\lambda \geq 5$ μm where the SiO_2 substrate is opaque, as shown below:[2,3]

$$R_\lambda = \rho_{12_{3=\infty}} + \frac{\rho_{31}\left(1-\rho_{12_{3=\infty}}\right)^2\tau^2}{1-\rho_{31}\rho_{12_{3=\infty}}\tau^2}, \tag{S3}$$

$$\tau = \exp\left(-\frac{4\pi\kappa d_s}{\lambda\cos\theta_s}\right). \tag{S4}$$

When $\lambda \geq 5$ μm, the second term in Eq. (S3) is omitted. In the above equations, subscripts 1, 2, 3 (or s) are for air, a-Si, and SiO_2, respectively, ρ_{31} represents the reflectance at the substrate-air interface, $\rho_{12_{3=\infty}}$ is the reflectance from the air to a semi-infinite substrate (medium 3) given by:

$$\rho_{12_{3=\infty}} = \left| r_{12} + \frac{t_{12}t_{21}r_{23}e^{i2\psi}}{1-r_{21}r_{23}e^{i2\psi}} \right|^2, \tag{S5}$$

where $\psi = \frac{2nd_2}{\lambda}\sqrt{n_2^2 - n_1^2\sin^2\theta_1}$ is the phase shift, and *t* and *r* are the Fresnel coefficients at the corresponding interface. Note τ counts for the path loss inside the substrate, κ is the extinction coefficient of SiO_2, $d_s = 1$ mm is the substrate thickness, and θ_s is the refractive angle inside the substrate which can be obtained through Snell's law. All the physical quantities used here are the averages for both TE and TM polarizations. In this model, the a-Si is assumed to be lossless to simplify the modeling. Ellipsometry would be needed to characterize both the refractive index and the extinction coefficient. It should be emphasized that a high-quality PECVD-deposited a-Si is nearly lossless in the 4 – 6 μm region, with a minimum extinction coefficient of nearly 0 and a

maximum of 0.037 around 5 μm, as reported in other literature.[4] The following formula is used for parametric modeling in the wavelength range of interest:[2]

$$n(\lambda) = \sqrt{n_0^2 + \frac{A}{\lambda^2}}, \tag{S6}$$

where the two fitting parameters are n_0 (the "DC" part) and A. Then, the fitted n is used to calculate the reflectance at different wavelengths and to match the measurement result. The results for the two samples after fitting are shown in Figure S2a. The final fitted parameters are shown in

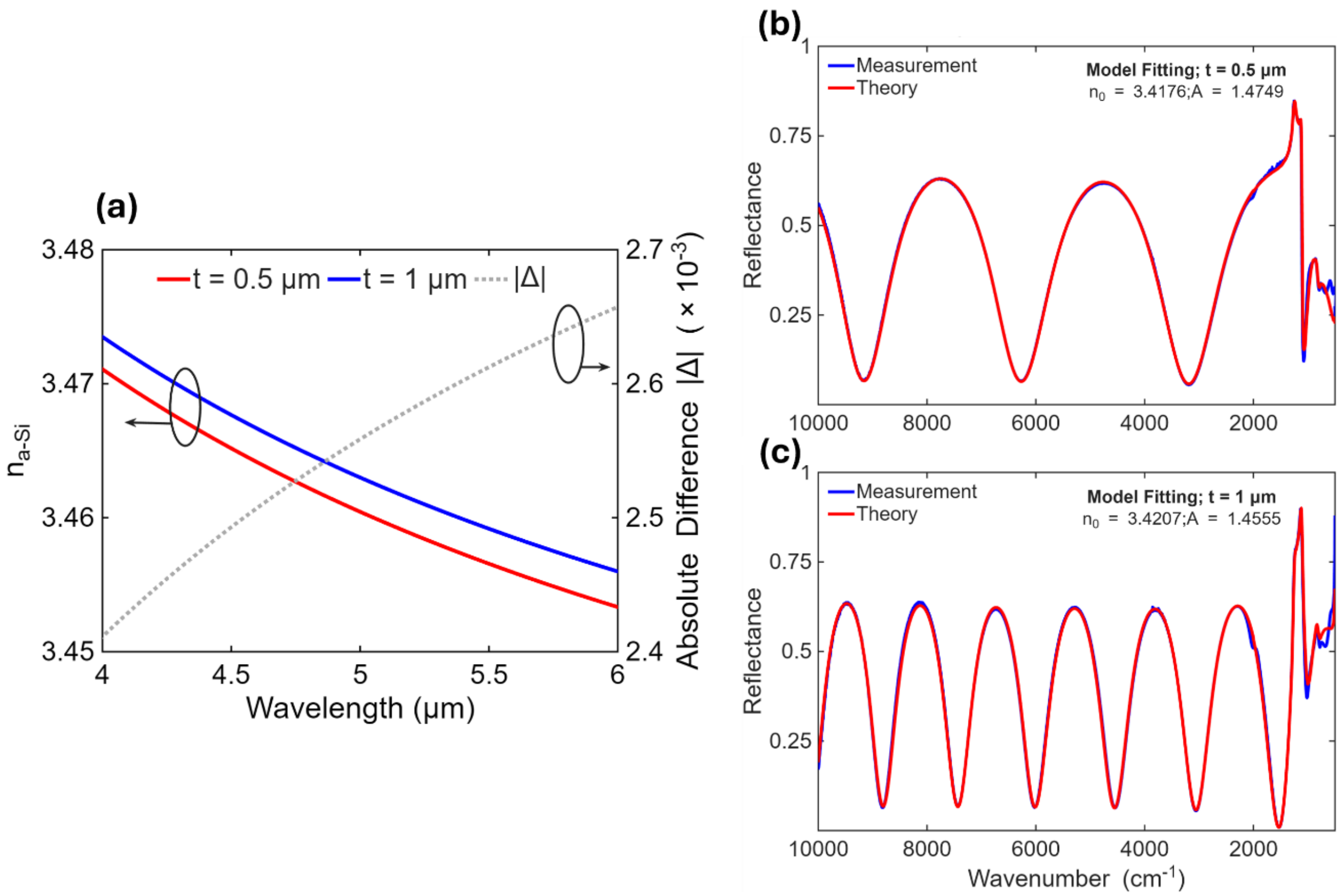

Figure S2. Refractive index measurement of PECVD a-Si. (a) The refractive index $n_{\mathrm{a-Si}}$ and the absolute difference, $|\Delta|$, between the two fitted models. (b) and (c) Measured reflectance for samples with different thicknesses of a-Si and parametric model fitted by using the parameters shown.

Figure S2b,c along with the comparison between the fitted model (theory) and the actual measurement results. The model agrees well with the experimental results. For the 0.5 μm sample, $n_0 = 3.42$ and $A = 1.47$, while for the 1 μm sample, $n_0 = 3.42$ and $A = 1.45$. Those parameters show great consistency with each other. In Figure S2a, the result refractive indices for the two samples are compared and the absolute difference is less than 2.7×10^{-3} in the wavelength range. The material data used in the simulation is obtained through the model described above. However, it is worth pointing out that the measurements on device and further numerical simulations show that the optical loss of a-Si depends on the quality of the film and its substrate, and therefore the actual refractive index can be slightly different from this model. The impact of the refractive index drift is discussed in the next section.

Effect of the fabrication imperfections

This section includes additional details on the numerical study of how fabrication uncertainties affect the device performance. Besides the width of the air gap, whose impact is discussed in the main text, the effects of top-grating thickness and finite optical loss of a-Si are reported here. Compared with the air-gap defect, these factors have relatively smaller effects on the overall device performance. However, they are still important for explaining several unexpected features observed in the experimental results, such as the emergence of double peaks and the increased emissivity baseline.

First, simulations were performed for the thickness variation of the top grating, which ranges from 900 to 1070 nm, as shown in Figure S3. Since the top grating has a period of 2 μm, which is more than twice smaller than the shortest wavelength of interest (4.6 μm), it can be considered as

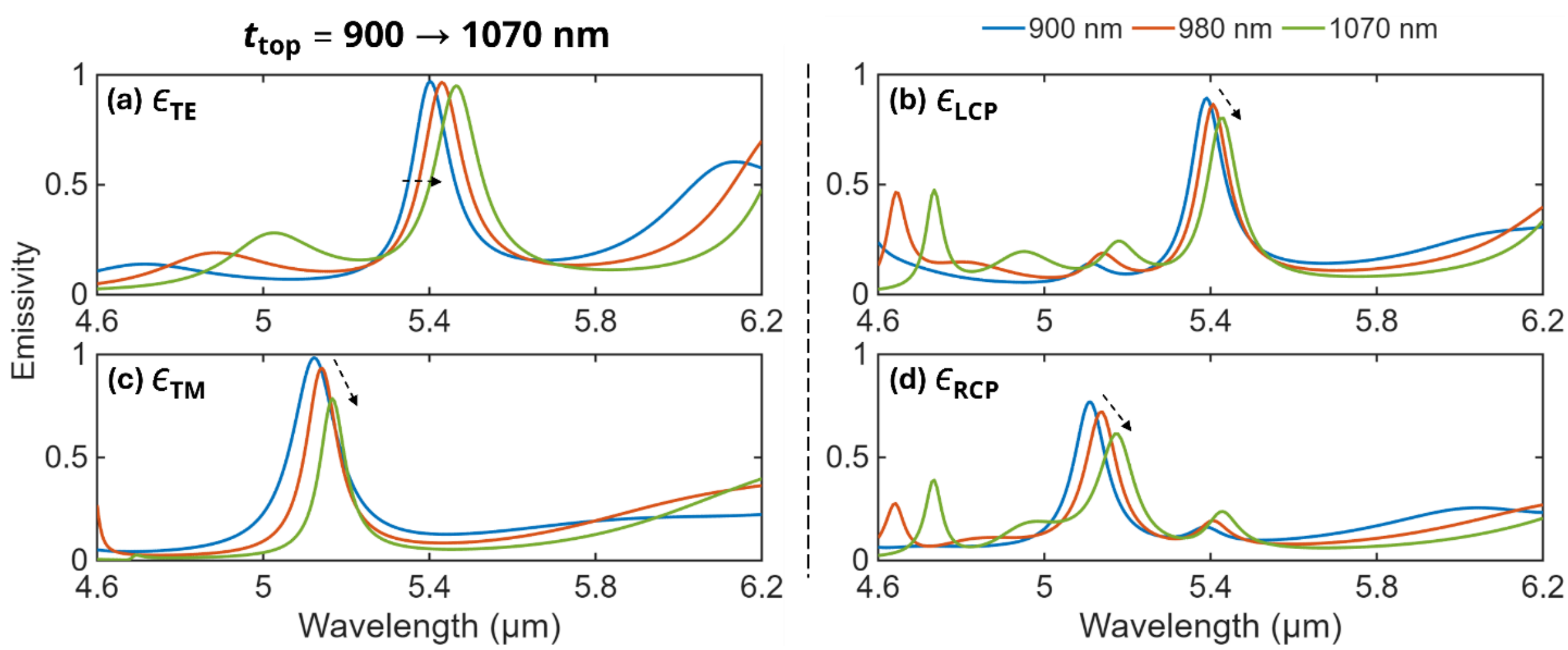

Figure S3. The impact of top layer grating thickness on the polarized emissivities of the device, for (a) TE, (b) LCP, (c) TM, and (d) RCP, obtained from RCWA simulations. The thickness ranges from 900 to 1070 nm. All other geometric parameters remain their original values.

an effective medium slab with in-plane anisotropy.[5] Therefore, slightly increasing the grating thickness shifts the resonance peaks towards longer wavelength, for both linear and circular polarizations. The secondary peaks around 4.7 μm also shift together with the main peak and consequently move into the wavelength range of interest. The increased top-grating thickness, together with the air gap defects in the bottom grating, contributes to the occurrence of the second peak near 4.8 μm in the experimental results.

In the ideal simulations, a-Si was assumed to be lossless. However, the optical loss of PECVD-deposited a-Si depends on the deposition conditions, and the actual loss in the fabricated a-Si film is higher than originally assumed and therefore cannot be ignored. By including a weak a-Si absorption ($\kappa = 0.03$) in the simulation, as shown in Figure S4, the device performance changes slightly and the polarization contrast is reduced, which better explains the degradation of the degree of polarization observed in the experiment. The measurement results in Figure 8 also

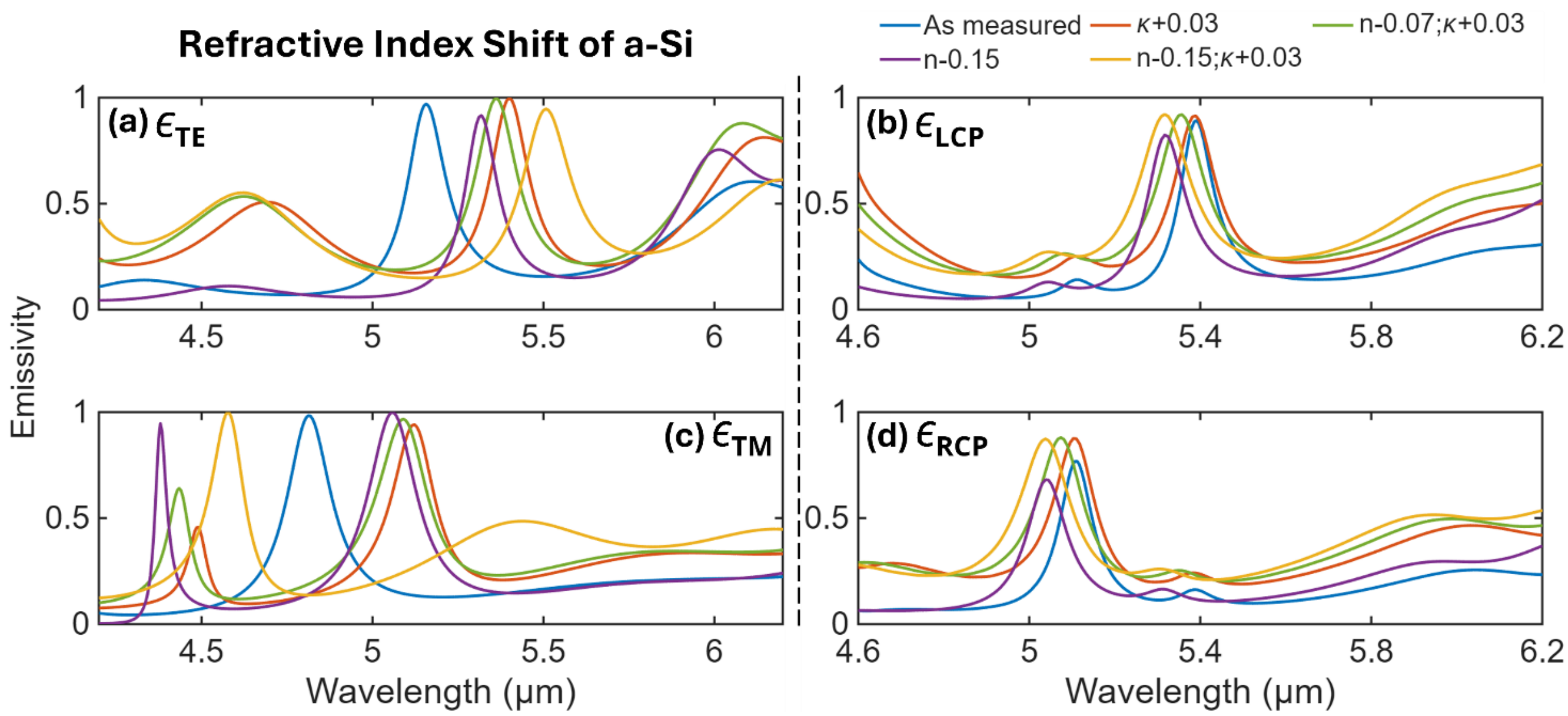

Figure S4. The impact of a-Si permittivity drift on the polarized emissivities of the device, for (a) TE, (b) LCP, (c) TM, and (d) RCP, obtained from FDTD simulations. The refractive index n at 5 μm ranges from 3.28 to 3.43, and the extinction coefficient κ ranges from 0 to 0.03. All other geometric parameters remain their original values.

show a raised emissivity floor approximately 0.2–0.3, higher than the value of around 0.1 predicted by the original design. This broadband weak absorption is likely due to material loss. Since the optical loss of SiO_2 has already been considered into simulation and the metals are highly conductive in this wavelength regime, the loss should mainly be attributed to the a-Si. Furthermore, the neighboring Cu is known to have a high diffusivity in Si as a positive ion, even at room temperature, which can increase the absorption in a-Si as a result of undesired doping.[6,7]

In addition, a secondary peak was observed experimentally near 5 μm. This feature mainly originates from a hybrid resonant mode of the top grating and the underlying oxide layer. The FDTD simulation has confirmed this, and the electric-field plot for the TE mode shown in Figure

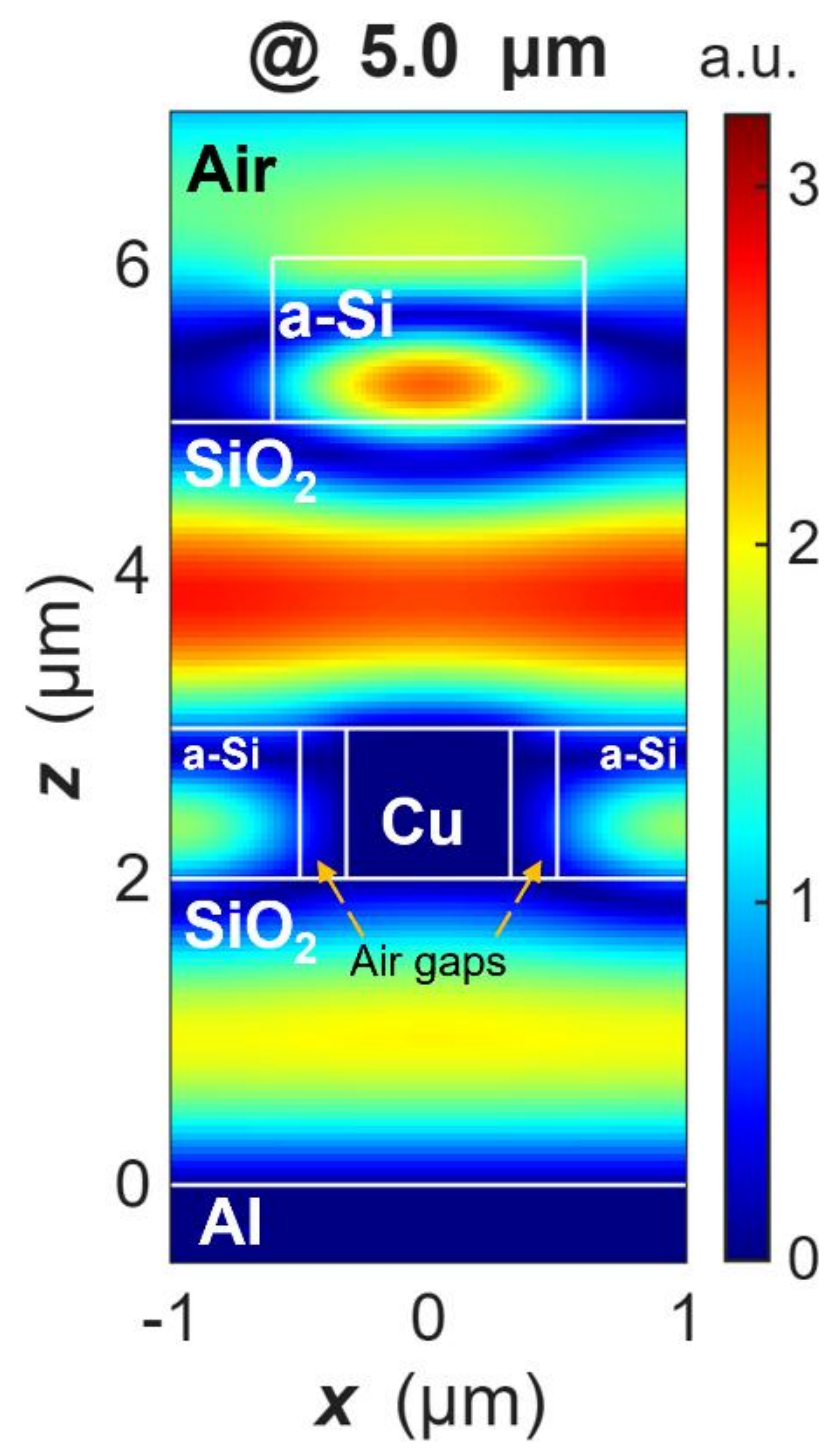

Figure S5. The electric-field plot, $|E_x|$, of the twisted-grating metasurface using fitted parameters for simulation. Air gap defect is incorporated into the simulation.

S5. In contrast, the resonance associated with the bottom grating and bottom oxide layer occurs near 5.5 µm and therefore does not strongly contribute to the emission peak observed around 5 µm.

<u>Optimization process</u>

The geometric parameters of the twisted-grating metasurface were optimized using a Python script consisting of two parts. The first part implements a particle swarm optimization algorithm using the open-source Scikit-Opt package[8]. This algorithm generates candidate device geometries within prescribed parameter ranges and evaluates their performance according to the polarization contrasts for LPs and CPs. The second part interfaces with Ansys Lumerical to automatically run the corresponding FDTD and RCWA simulations.

For each candidate geometry, an FDTD simulation was first performed for the 45° configuration. The reflectance spectra for LCP and RCP incident light were calculated, and the corresponding absorptivities were obtained using Kirchhoff's law: $\epsilon_i = \alpha_i = 1 - \rho_i$, where i stands for LCP or RCP. The wavelength at which the circular dichroism $|\epsilon_{\mathrm{LCP}} - \epsilon_{\mathrm{RCP}}|$ reaches its maximum was then identified. For the same geometry and wavelength, an RCWA simulation was subsequently performed for the 0° configuration to obtain the linearly polarized emissivities ϵ_{TE} and ϵ_{TM}.

Because the particle swarm optimization algorithm minimizes the objective function, the figure of merit (FoM) was defined in terms of the difference between one and the linear and circular polarization contrast:

$$\mathrm{FoM} = \sqrt{\frac{\delta\epsilon_{\mathrm{LP}}^2 + \delta\epsilon_{\mathrm{CP}}^2}{2}}, \tag{S7}$$

where $\delta\epsilon_{\mathrm{LP}} = 1 - |\epsilon_{\mathrm{TE}} - \epsilon_{\mathrm{TM}}|$ and $\delta\epsilon_{\mathrm{CP}} = 1 - |\epsilon_{\mathrm{LCP}} - \epsilon_{\mathrm{RCP}}|$. For an ideal linear polarized emitter, $\delta\epsilon_{\mathrm{LP}} = 0$, while for an ideal circularly polarized emitter, $\delta\epsilon_{\mathrm{CP}} = 0$. After iterating 200 rounds, the results are examined and slightly adjusted manually to satisfy the fabrication constraints.

Reference

(1) *HeatPad With 1" Diameter Hot Zone*. https://mhi-inc.com/product/gen2-heatpad-with-1-diameter-hot-zone/ (accessed 2026-03-17).

(2) Lee, B. J.; Zhang, Z. M.; Early, E. A.; DeWitt, D. P.; Tsai, B. K. Modeling Radiative Properties of Silicon with Coatings and Comparison with Reflectance Measurements. *Journal of Thermophysics and Heat Transfer* **2005**, *19* (4), 558–565. https://doi.org/10.2514/1.13596.

(3) Zhang, Z. M. *Nano/Microscale Heat Transfer*; 2nd edn., Springer Nature Switzerland AG: Cham, 2020. https://doi.org/10.1007/978-3-030-45039-7.

(4) Franta, D.; Nečas, D.; Zajíčková, L.; Ohlídal, I.; Stuchlík, J.; Chvostová, D. Application of Sum Rule to the Dispersion Model of Hydrogenated Amorphous Silicon. *Thin Solid Films* **2013**, *539*, 233–244. https://doi.org/10.1016/j.tsf.2013.04.012.

(5) Luque-González, J. M.; Sánchez-Postigo, A.; Hadij-ElHouati, A.; Ortega-Moñux, A.; Wangüemert-Pérez, J. G.; Schmid, J. H.; Cheben, P.; Molina-Fernández, Í.; Halir, R. A Review of Silicon Subwavelength Gratings: Building Break-through Devices with Anisotropic Metamaterials. *Nanophotonics* **2021**, *10* (11), 2765–2797. https://doi.org/10.1515/nanoph-2021-0110.

(6) Basu, S.; Lee, B. J.; Zhang, Z. M. Infrared Radiative Properties of Heavily Doped Silicon at Room Temperature. *J. Heat Transfer* **2009**, *132*, 023301. https://doi.org/10.1115/1.4000171.

(7) Hall, R. N.; Racette, J. H. Diffusion and Solubility of Copper in Extrinsic and Intrinsic Germanium, Silicon, and Gallium Arsenide. *J. Appl. Phys.* **1964**, *35* (2), 379–397. https://doi.org/10.1063/1.1713322.

(8) Guo, F. Guofei9987/Scikit-Opt, 2026. https://github.com/guofei9987/scikit-opt (accessed 2026-04-13).
